# The Frictional Vibration Attenuation of Rubber Utilizing a Groove on the Body

**DOI:** 10.3390/polym16121704

**Published:** 2024-06-14

**Authors:** Junhao Qu, Ruilin Wang, Rui Ren, Huabo He, Shuang Weng, Haibo Huang

**Affiliations:** 1 School of Mechanical Engineering and Mechanics, Ningbo University, No. 818, Fenghua Road, Ningbo 315211, China; k12345677p@outlook.com (J.Q.); 211081195@nbu.edu.cn (R.W.); renrui@nbu.edu.cn (R.R.); hehuabo@nbu.edu.cn (H.H.); 2111081203@nbu.edu.cn (S.W.); 2 Key Laboratory of Impact and Safety Engineering, Ministry of Education of China, Ningbo University, Ningbo 315211, China

**Keywords:** rubber, frictional vibration, groove, reduction, cube block

## Abstract

Frictionally induced vibrations in rubber are readily triggered due to their lower stiffness and higher elasticity. This study developed a numerical model to investigate the frictional vibration of a rubber block with a groove on its side surface against an aluminum disc. The results indicate that a backside groove (GB) on the block significantly enhances vibration attenuation, with a decay time 0.6 s faster than a non-grooved (NG) block, despite a potentially higher initial vibrational amplitude. In contrast, a frontside groove (GF) results in persistent frictional oscillations, with the steady-state time being similar for both GB and GF configurations. The underlying mechanism is attributed to the GB’s effectiveness in reducing the maximum energy imparted to the block initially, dissipating vibrational energy more swiftly, and distributing the contact stress more uniformly. The discrepancies in frictional forces between the conducted experiment and the simulation for the NG, GB and GF cases were 11.3%, 9.3% and 12.1%, respectively, quantitatively indicating the moderate precision of the results from the simulation. The insights gained from this study hold promise for enriching methods of mitigating vibrations arising from rubber friction.

## 1. Introduction

The soft–hard material friction pair is a classic friction pair frequently employed in applications such as tires, rubber rollers, belt pulleys, rubber seals, and stern bearings [1,2,3,4,5]. These pairs are crucial for vibration damping, friction enhancement, and sealing functions. Their performance significantly impacts the safety and reliability of both road transportation and aerospace equipment. Rubber, a prevalent soft material in friction pairs, possesses hyperelastic and viscoelastic [6,7] properties that introduce complex nonlinear and time-delay characteristics [8] into the friction system. These characteristics can potentially trigger friction-induced vibrations during contact. Consequently, the study of friction vibration in soft–hard material pairs has emerged as a focal point of research within both the scientific community and industry.

Friction–induced vibration phenomena vary with different contact configurations. It has been observed that decreasing the height and speed of rubber sheets in contact with glass can effectively reduce the vibration amplitude during sliding friction [9]. In the friction pair consisting of a shaft and a stern bearing, insufficient lubrication under conditions of low speed and heavy load can lead to increased and uneven local contact pressure, resulting in chatter, noise, and accelerated wear [10]. This suggests that variations in local contact pressure significantly influence the excitation of friction vibration. By incorporating rubber damping into a friction system with a hard–hard contact interface, it is possible to achieve lower contact stresses between friction pairs, thereby reducing the amplitude of friction vibration [11].

Research into the friction vibration of rubber–hard material interfaces lags behind that of hard–hard material interfaces. On one hand, the local contact behavior and the evolutionary mechanisms of friction vibration in rubber materials are not yet fully understood. The damping characteristics of rubber materials in friction vibration present greater complexity than those of rigid materials [11,12,13,14,15], leading to a paucity of systematic scientific foundations for addressing these issues. On the other hand, while there is a wealth of literature on frictional vibration, including studies on metallic materials [10,11,12,13,14,16], tire friction vibration [17], and lubrication-related friction vibration [18,19], the underlying mechanisms and control methods for frictional vibration in rubber materials are not as extensively documented. There is a shortage of targeted solutions for friction vibration problems of soft materials under specific conditions, such as dry friction and plate and shell structures. The local contact pressure variations at the interface significantly affect friction vibration excitation, and the behaviors of friction vibration differ between soft–hard and hard–hard friction interfaces. In contrast to previous research, examining the regulation of interface friction vibration in rubber materials from the perspective of the friction surface’s local area action mechanism offers a novel approach.

”Modifying material” and “modifying structure” are two pivotal strategies for achieving the suppression of friction-induced vibrations. Surface texture has emerged as a significant method for enhancing surface friction and reducing vibration. Jiliang Mo has proposed a method for regulating friction vibration noise at brake interfaces based on surface texture, particularly for hard–hard contact interfaces [20,21,22]. They accurately predicted the noise characteristics induced by friction vibration by manipulating the surface friction properties. On soft–hard material friction interfaces, textured surfaces exhibit improved tribological performance under water-lubricated conditions of stern bearings, achieving friction vibration suppression in the high-frequency range [23]. However, the surface texture on soft materials can deform irregularly due to the inherent material properties, which may affect the vibration suppression efficacy of the texture. Moreover, textured vibration suppression is not suitable for thin contact surfaces of soft materials. It is also important to recognize that friction vibrations can be influenced not only by features at the interface but also by the structural characteristics of the materials involved. Given the hyperelastic and viscoelastic properties of rubber, it is plausible that the appropriate rubber structure could affect vibrations at the frictional interface. Nevertheless, the specific ways in which rubber structure impacts frictional vibration, and the extent of this influence, remain unclear.

In terms of “modifying structure”, it has been observed that the addition of graphene sheets to composite materials can reduce the friction coefficient due to their excellent lubricating properties, thereby suppressing friction vibration under water-lubricated conditions [24]. Carbon tubes and nano zinc oxide have been shown to enhance both the damping and deformation properties of high-molecular-weight polyethylene, and their synergistic effects can reduce the friction vibration amplitude of polymers [25]. Currently, this method is primarily employed under water-lubricated conditions and is associated with high material preparation costs and complex processing requirements.

In this study, a novel methodology is introduced to mitigate frictional vibration in the contact pair between rubber and aluminum materials, focusing on the approach of “modifying structure”. The research employs the finite element (FE) method to examine the effects of texturing a rubber block with a groove on its flank, rather than on the contact face. The investigation aims to understand the groove’s impact on vibration amplitudes, contact stress, and friction force. Through this analysis, the optimal structure for vibration suppression is identified and subsequently validated by experimental tests. The methodology proposed in this study is expected to provide guidance and expand the range of techniques available to suppress frictional vibration.

## 2. Methodology

**Figure 1:**
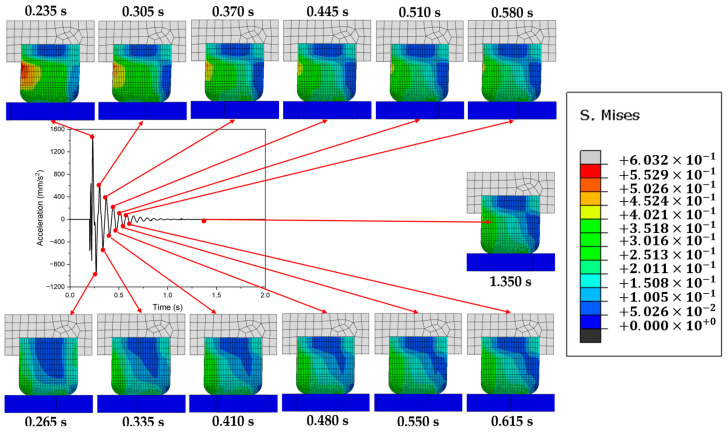
Evolution of the distribution of stress during frictional vibration.

Figure 1 illustrates the friction-induced vibrations occurring as a rubber specimen slides against an aluminum plate, utilizing the FE method. The aluminum plate was tightly fixed, while the rubber was moved from left to right. Snapshots of the distribution of stress at the peaks of each cycle were captured. It was noted that as the vibration amplitude climaxed during a cycle, a stress concentration zone emerged on the backside of the rubber, opposite the direction in which the rubber block moves. This effect was particularly pronounced during the initial cycles. This observation sparked the hypothesis that carving a groove in the area of stress concentration might dissipate and scatter the stress, thereby mitigating friction-induced vibrations. This notion serves as the impetus for the current investigation into the effects of grooving on the progression of frictional vibrations. Additionally, the FE model employed in this study will be presented in the following section.

## 3. Modeling

### 3.1. FE Model

**Figure 2:**
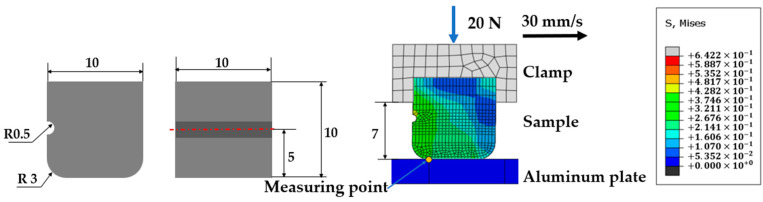
The shape and dimension of a rubber block.

Figure 2 depicts the geometry and dimensions of the rubber block, which features of two chamfers on its front and back sides, each with a radius of 3 mm. The opposing surface, known as the counterpart, is crafted from aluminum. The FE model of the rubber block in contact with the aluminum counterpart is displayed in Figure 2. A groove, intended to alleviate stress concentration during sliding, has been precision-cut into the rubber block. This groove has a diameter of 1 mm and is positioned 5 mm from the contact interface.

In the simulation, the C3D8R element is utilized, which is a three-dimensional, eight-node hexahedral element known for its robust deformation capabilities, excellent convergence properties, and computational stability [26]. The rubber block and the aluminum plate are discretized into 12,150 and 440 elements, respectively. The node counts for the rubber block and the aluminum plate are 13,754 and 693, respectively. To enhance the accuracy of the simulation, the meshes near the contact region as well as the position where the groove exists were locally refined, as depicted in Figure 2.

For clarity, the position of the groove is defined. Additionally, the right side of the block is designated as the front. When the groove is located at the back of the block, as illustrated in Figure 2, it is referred to as the GB (groove at back) configuration. Conversely, the GF (groove at front) configuration indicates that the groove is positioned at the front of the block, while the NG (no groove) case denotes the absence of a groove on the rubber block.

The measurement point, as depicted in Figure 2, is situated on the contact surface of the rubber block. All the results presented in Section 4 are based on data recorded at this specific point.

**Figure 3:**
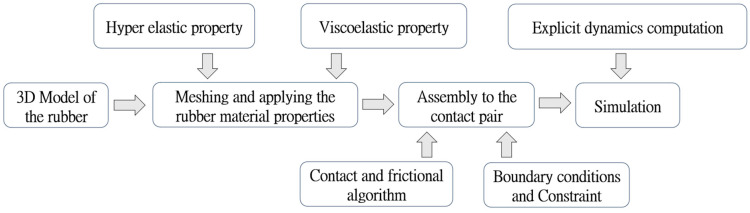
Processing flowchart of the FE model.

The processing flowchart of the FE model is illustrated in Figure 3. A detailed description for the factors, e.g., materials properties and contact algorithm will be given in the following section.

### 3.2. Material Features

Accurate simulation relies heavily on capturing material properties correctly. Rubber materials exhibit both hyperelastic and viscoelastic characteristics, which are typically described by various constitutive equations. In this study, the Mooney–Rivlin model and the Maxwell model are used to characterize the hyperelastic and viscoelastic properties of the rubber, respectively.

#### 3.2.1. Mooney–Rivlin Model

The Mooney–Rivlin model [26] can be written in a simple form that can describe medium and small deformations precisely:

(1) $$U=c_{10}\left(I_{1}-3\right)+c_{01}\left(I_{2}-3\right)$$

where U is the strain energy potential;$\;I_{1},\;I_{2}$ are the first and second deviatoric strain invariants; and $c_{10},\;c_{01}$ are the material parameters. The two parameters of the Mooney–Rivlin constitutive model were $c_{10}$ = 2, and $c_{01}$ = 0.5.

#### 3.2.2. Generalized Maxwell Model

**Figure 4:**
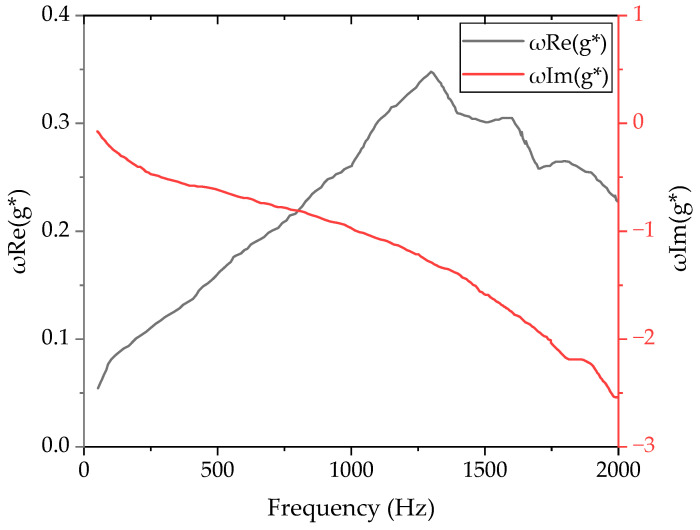
Real part (*ωRe*(g*)) and imaginary part (*ωIm*(g*)) of the rubber’s viscoelastic characteristics.

The generalized Maxwell model expands on this by incorporating multiple Maxwell elements in parallel with an ideal spring. In the generalized Maxwell model [27], after applying Fourier transformation to the stress(*σ*)–strain(*ε*) relationship, the shear storage modulus $G_{s}\left(\omega \right)$, and shear loss modulus $G_{L}\left(\omega \right)$ can be defined by specific formulas:

(2) $$G_{s}\left(\omega \right)=K+\overset{N}{\underset{i=1}{\sum }}\frac{{K_{i}}^{2}{c_{i}}^{2}\omega^{2}}{{K_{i}}^{2}+{c_{i}}^{2}\omega^{2}}$$

(3) $$G_{L}\left(\omega \right)=\overset{N}{\underset{i=1}{\sum }}\frac{{K_{i}}^{2}{c_{i}}^{2}\omega }{{K_{i}}^{2}+{c_{i}}^{2}\omega^{2}}$$

where K is the elastic modulus, ω is the frequency, and $K_{i}$ (i = 1, 2 … *N*) and $c_{i}$ (*i* = 1, 2 … *N*) are the elastic modulus and damping in each elastic element, respectively. The equations could not be used directly with commercial software [28]. In Abaqus, a material’s viscoelastic characteristics can be defined using the normalized shear relaxation function in the frequency domain, represented by the real part ($\omega Re\left(g^{*}\right)$) and the imaginary part ($\omega Im\left(g^{*}\right)$) of the function:

(4) $$\omega Re\left(g^{*}\right)=\frac{G_{L}\left(\omega \right)}{G_{\infty }}$$

(5) $$Im\left(g^{*}\right)=1-\frac{G_{s}\left(\omega \right)}{G_{\infty }}$$

where $G_{\infty }$ is the semi-static shear modulus, which is related to the material’s hyperelastic parameters. Figure 4 shows the real part ($\omega Re\left(g^{*}\right)$) and the imaginary part ($\omega Im\left(g^{*}\right)$) of the rubber, which was identified by the experiment.

### 3.3. Contact Algorithm

FE contact analysis encompasses several methods, including Lagrange multipliers, the penalty method, hybrid and mixed methods, and direct constraints [28]. Each of these methods has its own advantages and limitations, and the choice of method often depends on the specific requirements of the analysis and the computational resources available.

In this study, the penalty approach was selected to model the contact interaction, which is akin to using nonlinear springs between the contacting bodies [28]. It allows for some penetration, the extent of which is governed by the penalty constant or function. The penalty method can be expressed as follows:

(6) $$F_{normal}=K_{normal}\times P_{penetration}\;$$

where $F_{normal}$ is the contact stress, $K_{normal}$ is the contact stiffness between the two contact bodies, and $P_{penetration}$ is the penalty parameter.

This method is relatively straightforward to implement and is widely used in explicit dynamic analyses, which allows for a practical balance between computational efficiency and the accuracy of contact simulation.

### 3.4. Friction Algorithm

Coulomb friction model was employed in this study to compute the frictional behavior between the hard and soft materials, which is also the most popular friction model. The Coulomb model can be characterized by [28]

(7) $$\sigma_{t}<\mu \sigma_{n}\left(stick\right)\;and\;\sigma_{t}=-\mu \sigma_{n}\cdot t\left(slip\right)$$

(8) $$t=\frac{v_{r}}{v_{r}}$$

where $\sigma_{t}\;$ is the friction stress, $\sigma_{n}\;$ is the normal stress, μ is the friction coefficient, *t* is the tangential vector in the direction of the relative velocity, and $v_{r}\;$ is the relative sliding velocity.

## 4. Results and Discussion

### 4.1. With and without Groove

**Figure 5:**
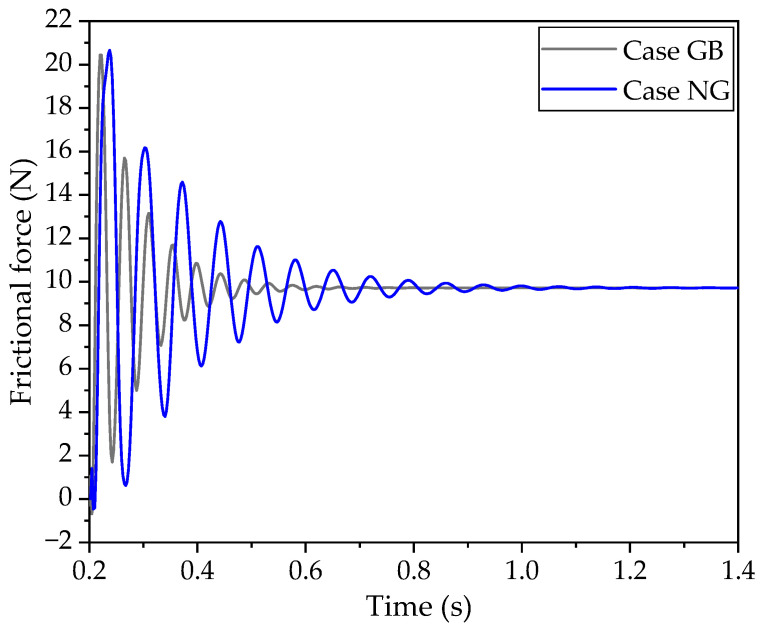
Comparison of the frictional force.

Figure 5 depicts the frictional forces for the GB and NG cases, which represent cases with and without a groove, respectively. It is evident that the frictional force in the GB case is significantly lower than that in the NG case, particularly after the second and subsequent periods. Additionally, the rate at which the amplitude of the frictional force tends to a steady state in the GB case is approximately 0.6 s faster than in the NG case. This indicates that the presence of the groove significantly enhances the damping ability, effectively reducing the frictional force.

**Figure 6:**
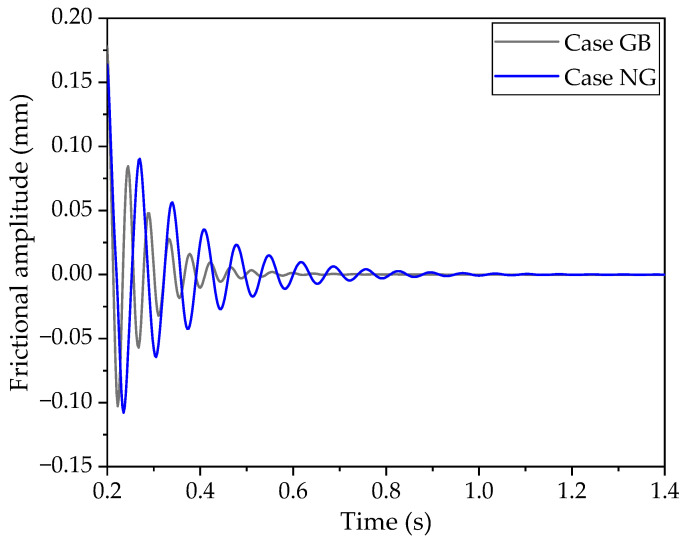
Vibrational amplitudes of the block with- and without groove.

Figure 6 compares the vibrational amplitudes for the scenarios with and without a groove. It is observed that the amplitude in the NG case is notably higher than in the GB case. Similar to the frictional force, the vibrational amplitude in the GB case decays at a faster rate than in the NG case. Furthermore, the deformation of the block in the NG case is approximately 0.05 mm greater than in the GB case in steady state. This suggests that the presence of the groove reduces the block’s stiffness, allowing for an increased deformation.

### 4.2. Groove on Front and Back Side

**Figure 7:**
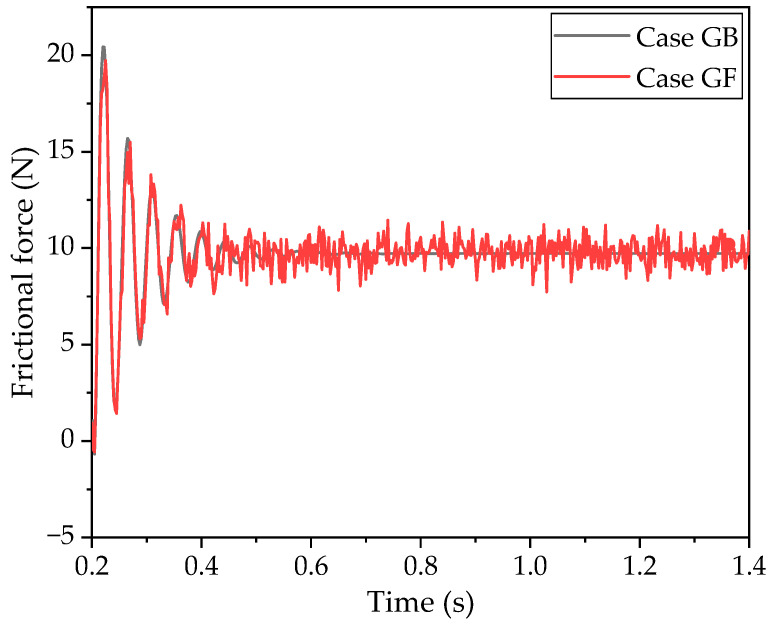
Frictional force of the block moving forward and opposite directions.

Figure 7 represents the frictional forces in the tangential direction for the GB and GF cases, which exhibit cases with a groove on the front and back side, respectively. It is observed that the frictional force decreases over time in both scenarios. In the steady state, the frictional force is approximately 10 N for both configurations. In the GB case, the frictional force stabilizes at around 0.7 s. However, in the GF case, oscillations persist around the 10 N mark, with an oscillation amplitude of roughly 3 N.

**Figure 8:**
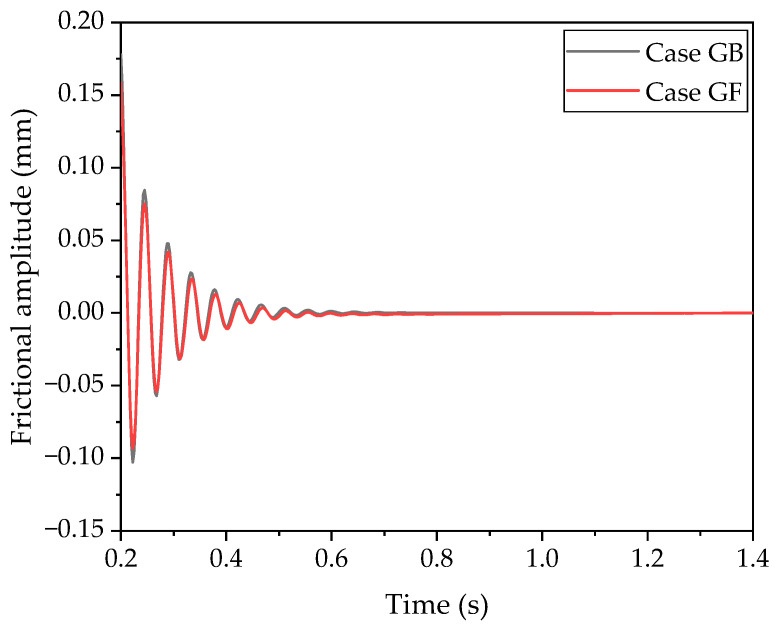
Vibrational amplitudes of the block moving in forward and opposite directions.

Figure 8 depicts the vibrational amplitudes in the tangential direction for the GB and GF cases. It is evident that the vibration amplitudes decay progressively in both cases. The maximum amplitude is roughly 0.15 mm for both configurations. The frictional amplitudes in both directions are nearly identical, and the attenuation rate in the GB case is very similar. This suggests that the influence of the direction change on vibrational amplitude is not significant.

### 4.3. Distribution of Stress

**Figure 9:**
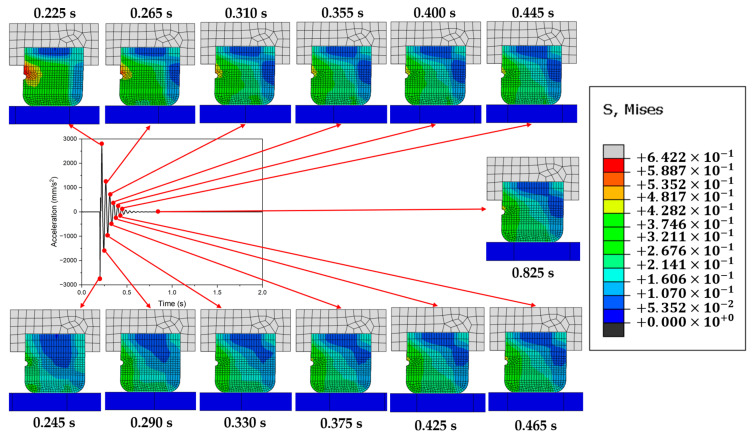
Distribution of stress from the side profile with time variation.

Figure 9 displays the distribution of stress in the block side profile over time for the GB case, with snapshots captured at the time when the maximum and minimum amplitudes appear within each period. It is evident that overall, the stress at the front of the block is less than that at the back. Notably, when the vibrational amplitude reaches its positive maximum, stress concentrations in the rubber are observed around the groove. Conversely, when the amplitude is at its negative maximum, the distribution of stress is more even, reminiscent of the findings presented in Figure 1. The vibration period is approximately 0.044 s in the GB case. Although in both GB and NG cases, the stress around the groove is the highest in the rubber when the vibrational amplitude reaches its positive maximum, the period of the vibration in the GB case is approximately 0.024 s shorter than that in the NG case.

**Figure 10:**
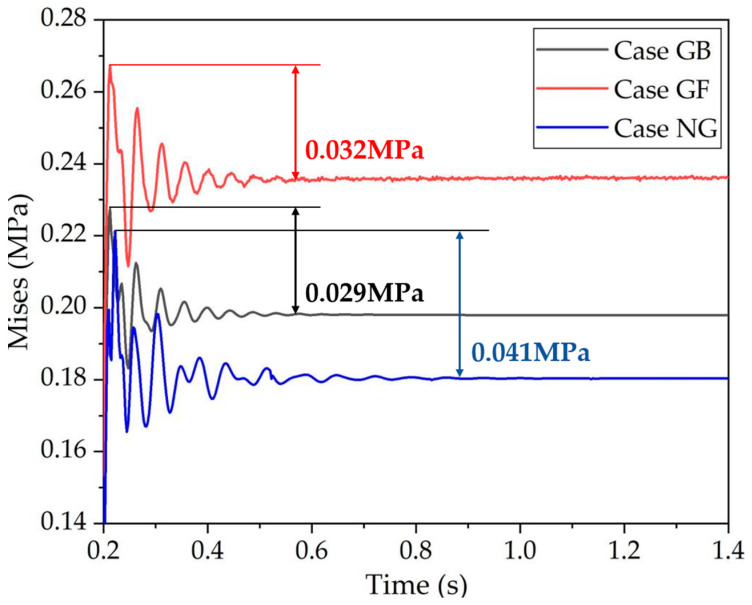
Stress at the measuring point with time variation.

Figure 10 illustrates the stress variation over time. It is evident that in the steady state, the stress is the highest in the GF case, followed by the GB and NG cases. However, the stress in the GB case reaches a steady state first, while the stress in the NG case is the last to stabilize. Similar to the vibrational amplitude in the GF case, the stress in this configuration also exhibits continuous oscillation. The differences between the maximum stress and the steady-state stress are 0.041 MPa, 0.029 MPa, and 0.032 MPa for the NG, GB, and GF cases, respectively. The smallest difference is observed in the GB case, suggesting that the presence of a groove at the back of the rubber block accelerates the decaying of vibrational energy.

**Figure 11:**
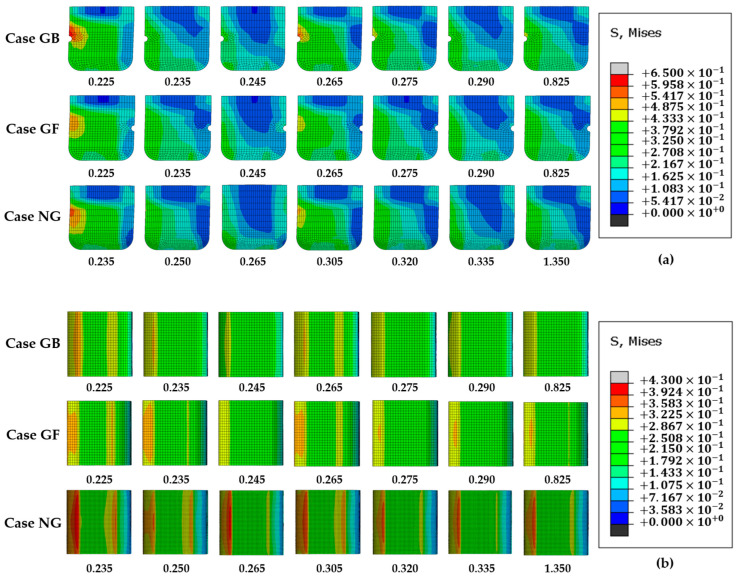
Distribution of stress in contact and from a side view. (**a**) distribution of stress from a side view; (**b**) distribution of stress in the contact patch.

Figure 11 presents the distribution of stress on the contact patch and from a side view for all three cases over time. As seen in Figure 11a, due to the direction of movement, the higher and lower stress regions are located in the top left and bottom right areas of the blocks, respectively, across all cases. Stress concentration zones are evident in the back top region of the block. Figure 11b reveals that in the NG case, there are two stress concentration regions in the front and back contact patches of the block, which lack grooves. In contrast, the stress is more evenly distributed in the GB case, in which the groove is positioned at the back of the block.

### 4.4. Discussion

Considering Figure 5, Figure 6, Figure 10 and Figure 11, it is apparent that both the frictional force and vibrational amplitudes are swiftly attenuated when a groove is introduced to the back side of the block. Additionally, the presence of the groove results in a more uniform distribution of contact stress in the GB case, as illustrated in Figure 11b. This more even distribution of stress may also contribute to the faster rate of weakening, according to the authors’ interpretation. Furthermore, although the stress in the GB case is moderate in the steady state, the initial maximum stress amplitude is the smallest, while the stress amplitude in the NG case is the greatest. This indicates that the vibrational energy in the GB case is lower than in the other two cases, suggesting that the placement of the groove on the back side of the block is effective in dissipating vibrational energy and reducing frictional forces.

Considering Figure 7, Figure 8, and Figure 11, collectively, the impact of the groove’s position on the frictional force and vibration amplitude is also related to the direction of sliding. Oscillations are observed in both the frictional force and stress when the groove is on the front, aligning with the direction of movement. Conversely, when the groove is on the back of the block, the rubber does not exhibit oscillatory behavior. The distribution of stress across the contact area is notably more uneven when the block lacks a groove. In essence, the presence of a groove tends to facilitate a more even distribution of stress within the contact area.

## 5. Experimental Verification

### 5.1. Sample Preparation

**Figure 12:**
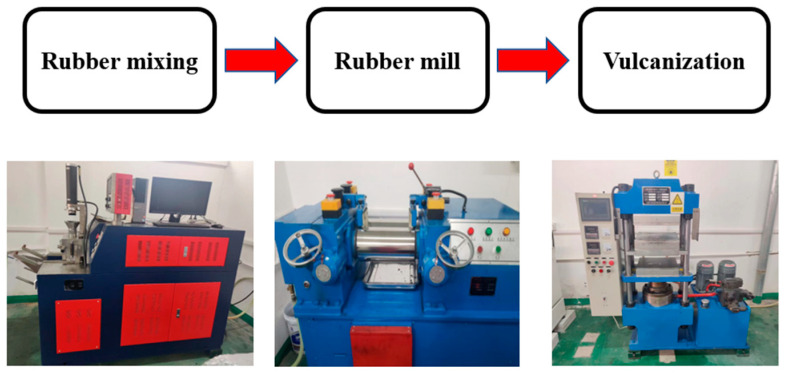
Sample preparation process.

The sample preparation process is detailed in Figure 12. Rubber and additives are thoroughly mixed to create a uniform blend, a crucial step in ensuring that rubber products exhibit consistent performance and quality. The rubber mixing mill is instrumental in blending and plasticizing the raw materials, providing the calendered plastic products with a molten material that is both uniformly mixed and plasticized. The final stage involves vulcanization, where auxiliary materials are added to induce chemical and physical changes in the rubber, enhancing its properties and rendering it suitable for practical use. Rubber vulcanization is a process that requires precise control of pressure, temperature, and time, as these factors must be carefully managed to meet the specific performance requirements of the rubber products. The sample composition and ratios include natural rubber/butadiene rubber (NR/BR) at a ratio of 80/20 parts per hundred rubber (phr), along with 20 parts carbon black, 2 parts stearic acid, 5 parts zinc oxide, and 3 parts sulfur, among other components.

### 5.2. Test Rig and Test Samples

**Figure 13:**
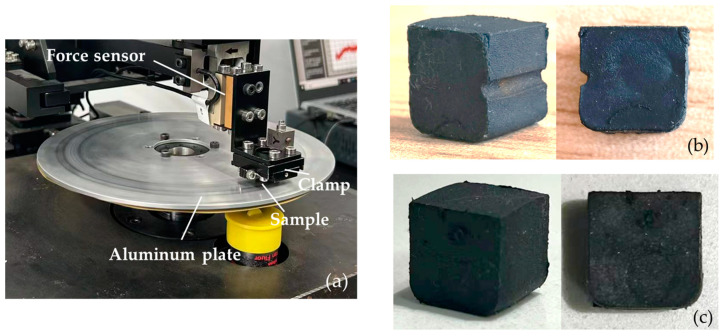
Test rig and the rubber samples. (**a**) The test rig; (**b**) rubber block with groove; (**c**) rubber block without groove.

The test rig depicted in Figure 13a comprises several key components, including a beam, a clamping mechanism to secure the rubber block, and a rotary disc made of aluminum. Two force sensors are mounted to measure the frictional force in the x-direction and the vertical force in the y-direction, respectively. The rubber blocks with and without a groove are illustrated in Figure 13b,c, respectively.

Prior to initiating the experiment, the rubber cube is affixed within a fixture, ensuring that it contacts the aluminum plate. A system of weights and crossbeams is employed to press the rubber block firmly against the aluminum metal plate. The pressure exerted on the rubber block is gauged by a mechanical sensor positioned above the fixture, while the weight is adjusted to achieve a normal load of 20 N on the rubber cube. The linear velocity at the point of contact between the aluminum plate and the rubber block is set to 30 mm/s. The experiment involves recording the changes in the friction curve of the rubber block until the friction force stabilizes for a duration of 10 s, at which point the experiment is concluded.

### 5.3. Experiment Results

**Figure 14:**
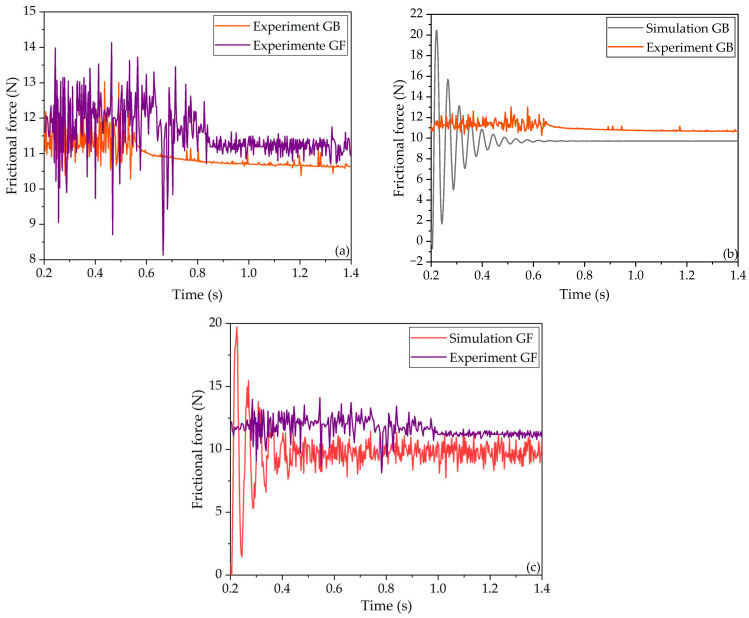
Comparison of the frictional force in the GB and GF cases. (**a**) Comparison of the experimental data between the GB and GF cases; (**b**) Data comparison of the GB case between the simulation and the experiment; (**c**) Data comparison of the GF case between the simulation and the experiment.

**Figure 15:**
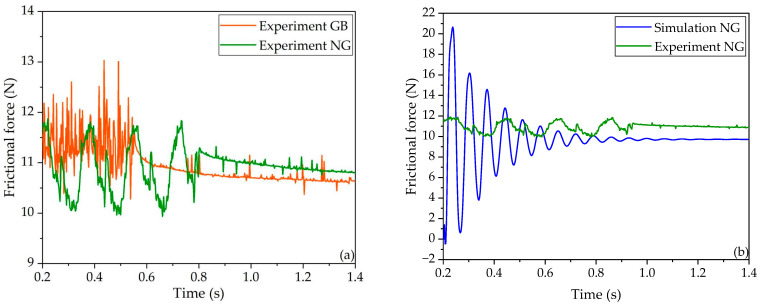
Comparison of the frictional force in the GB and NG cases. (**a**) Comparison of the experimental data between the GB and NG cases; (**b**) comparison of the data between the simulation and the experiment in the NG case.

To quantify the verification, two sets of experiments were conducted. The first set involved configurations with grooves on the front and back, respectively, while the second set compared scenarios with and without a groove. Figure 14 and Figure 15 both display the comparison between the simulation and experiment in two cases. The comparison between the simulation and experimental data indicates a reasonable level of agreement, which serves to validate the accuracy of the simulation. It is important to note that any discrepancies between the simulation and experimental results can be attributed to the ideal conditions under which the simulation was conducted. In real-world scenarios, factors such as the rigidity of the test setup and the purity of the materials can introduce variables that may influence the outcomes.

As depicted in Figure 14a, the time taken to reach a steady state is less in the GB case than in the GF case. Additionally, in both simulation and experiments, the vibrations continue in the GF case to a steady state and are depressed in the GB case, qualitatively indicating good agreement. Furthermore, as depicted in Figure 14b,c, the frictional forces measured for the GB and GF cases were 10.6 N and 11.2 N when the vibration was suppressed or in a steady state, respectively. The discrepancies in frictional forces between the experimental results and the FE simulations for the GB and GF cases were 9.3% and 12.1%, respectively, quantitatively implying a moderate agreement between the simulation and the experimental data in both the GB and GF cases.

Similarly, as illustrated in Figure 15a, the depression time for the GB case is faster than that for the NG case, which qualitatively implies good agreement with the simulation data. As depicted in Figure 15b, the discrepancy between the experimental results and the simulation for the frictional force of the NG case was 11.3%, quantitatively indicating moderate agreement between the simulation and the experimental data.

Considering both Figure 14 and Figure 15, the comparison confirms that the grooves placed at the back of the block exhibit attenuation capabilities for frictional vibration compared to both the front placement and absence of grooves. The findings from the experimental analysis align quantitatively with those from the FE simulation, providing validation of the accuracy of the results obtained in the FE simulation.

## 6. Conclusions

The paper introduces an FE model developed to explore the impact of grooves on a block body on frictional vibration, with experimental validation conducted to verify the findings. The key conclusions derived from this research include the following:

(1) The influence of the groove on the rubber frictional vibration is significant. Placing the groove on the back of the block opposite the direction of movement accelerates the decaying of vibrational energy. Additionally, the presence of the groove leads to a more even distribution of contact stress, which may contribute to the observed reduction in frictional vibration.
(2) The position of the groove and the direction of sliding are critical factors affecting frictional vibration. A groove located on the back of the block opposing the direction of movement is more effective in attenuating frictional vibration than a groove on the front of the block.
(3) The groove positioned on the back of the block opposite the direction of movement is the optimal choice for suppressing frictional vibration. This is due to the mechanism by which the groove acts to reduce the maximum initial energy in the block, accelerating the dissipation of vibrational energy and promoting a more even distribution of contact stress.

There are numerous parameters that determine the shape of rubber tread. The influence of the groove’s position on the rubber, its dimensions, and the clamping position on the rubber will be further investigated to improve the accuracy of this method in the near future.

## Data Availability

The data presented in this study are available on request from the corresponding author due to technical limitations.

## Nomenclature

Symbol	Description
U	Strain energy potential
$I_{1}$	First deviatoric strain invariants
$I_{2}$	Second deviatoric strain invariants
$c_{10}$	Material parameters
$c_{01}$	Material parameters
σ	Stress
ε	Strain
$G_{s}\left(\omega \right)$	Shear storage modulus
$G_{L}\left(\omega \right)$	Shear loss modulus
K	Elastic modulus
ω	Frequency
$K_{i}$ (i = 1, 2 … *N*)	Elastic modulus in each elastic element
$c_{i}$ (*i* = 1, 2 … *N*)	Damping in each elastic element
$\omega Re\left(g^{*}\right)$	Real part of the normalized shear relaxation function
$\omega Im\left(g^{*}\right)$	Imaginary part of the normalized shear relaxation function
$G_{\infty }$	Semi-static shear modulus
$F_{normal}$	Contact stress
$K_{normal}$	Contact stiffness between the two contact bodies
$P_{penetration}$	Penalty parameter
$\sigma_{t}$	Friction stress
$\sigma_{n}$	Normal stress
μ	Friction coefficient
*t*	Tangential vector in the direction of the relative velocity
$v_{r}$	Relative sliding velocity
GB	Rubber block with groove at back
GF	Rubber block with groove at front
NG	Rubber block without groove
